# Shear margins in upper half of Northeast Greenland Ice Stream were established two millennia ago

**DOI:** 10.1038/s41467-024-45021-8

**Published:** 2024-02-08

**Authors:** Daniela Jansen, Steven Franke, Catherine C. Bauer, Tobias Binder, Dorthe Dahl-Jensen, Jan Eichler, Olaf Eisen, Yuanbang Hu, Johanna Kerch, Maria-Gema Llorens, Heinrich Miller, Niklas Neckel, John Paden, Tamara de Riese, Till Sachau, Nicolas Stoll, Ilka Weikusat, Frank Wilhelms, Yu Zhang, Paul D. Bons

**Affiliations:** 1 Alfred Wegener Institute Helmholtz Centre for Polar and Marine Research, Bremerhaven, Germany; 2 Department of Geosciences, Tübingen University, Tübingen, Germany; 3 Now at ATLAS ELEKTRONIK GmbH, Bremen, Germany; 4 Niels Bohr Institute, Physics of Ice, Climate and Earth, University of Copenhagen, Copenhagen, Denmark; 5 Center for Earth Observation Sciences, University of Manitoba, Winnipeg, Canada; 6 Now at Laboratoire de Géologie de Lyon: Terre, Planètes, Environnement (LGL-TPE), ENS Lyon, Université Claude Bernard Lyon 1, CNRS, Villeurbanne, France; 7 University of Bremen, Bremen, Germany; 8 College of Earth Science, Chengdu University of Technology, Chengdu, China; 9 Geoscience Centre, University of Göttingen, Göttingen, Germany; 10 GEO3BCN, CSIC, Lluís Solé Sabarís s/n, 08028 Barcelona, Spain; 11 Center for Remote Sensing and Integrated Systems (CReSIS), University of Kansas, Lawrence, KS USA; 12 Now at Department of Environmental Sciences, Informatics and Statistics, Ca’Foscari University Venice, Venice, Italy; 13 School of Earth Science and Resources, China University of Geosciences (Beijing), Beijing, China

**Keywords:** Cryospheric science, Structural geology

## Abstract

Only a few localised ice streams drain most of the ice from the Greenland Ice Sheet. Thus, understanding ice stream behaviour and its temporal variability is crucially important to predict future sea-level change. The interior trunk of the 700 km-long North-East Greenland Ice Stream (NEGIS) is remarkable due to the lack of any clear bedrock channel to explain its presence. Here, we present a 3-dimensional analysis of the folding and advection of its stratigraphic horizons, which shows that the localised flow and shear margins in the upper NEGIS were fully developed only ca 2000 years ago. Our results contradict the assumption that the ice stream has been stable throughout the Holocene in its current form and show that upper NEGIS-type development of ice streaming, with distinct shear margins and no bed topography relationship, can be established on time scales of hundreds of years, which is a major challenge for realistic mass-balance and sea-level rise projections.

## Abstract (web summary)

The flow of ice streams leaves traces in the stratigraphy of the ice sheets. Made visible by radar, they reveal the history of the upper North East Greenland Ice Stream. The ice stream is found to have existed in its current form for only about the last 2000 years.

## Introduction

**Fig. 1:**
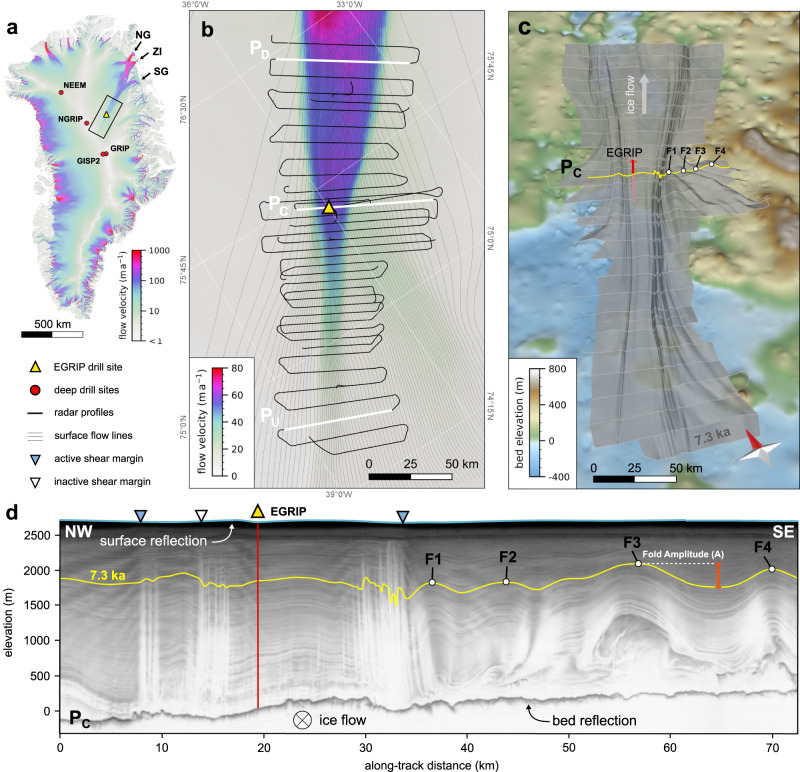
Area and outline of the radar survey. **a, b** Maps of the survey area and radar lines with ice flow velocity^11^. NG: Nioghalvfjerdsfjorden Glacier, ZI: Zachariae Isstrøm, SG: Storstrømmen Glacier. Radar profile, P_C_ is shown in panel (**d**) and in Figure 2b, P_U_ and P_D_ are shown in Fig. 2a,c. C stands for Centre, D for Downstream and U for Upstream. **c** 7.3 ka BP isochrone horizon as a shaded relief above bedrock^64^ (10 times vertical exaggeration). F1-F4 indicate the location of fold anticlines also highlighted in the radar profile P_C_ in panel (**d**). The location of P_C_ is indicated by a yellow line. **d** Profile P_C_ across Northeast Greenland Ice Stream (NEGIS) in the centre of the survey area. F1-F4 indicate the anticlines highlighted in panel **c**.

Most of the discharge of ice into the oceans takes place by solid ice flow via ice streams^1,2^. These are river-like zones where ice flow is significantly faster than in their surroundings, assumed to be triggered by either bedrock properties^3,4^, enhanced sliding^2,5^, and/or by deformation mechanisms leading to shear localization^6^. The most conspicuous one in Greenland is the Northeast Greenland Ice Stream (NEGIS; Fig. 1a), which extends for about 700 km inland from its outlets in Northeast Greenland, and its catchment covers 17 % of the ice sheet area^7^.

NEGIS reaches up to the central ice divide (Fig. 1a), but while the gates in the coastal mountain range in northeast Greenland clearly determine the location of the coastal outlet, its course in the interior of the ice sheet appears not to be constrained by bed topography^8–10^. The flow velocity increases from about 3 m yr^−1^ close to the divide to 55 m yr^−1^ at the EGRIP (East Greenland Ice core Project) drilling camp^11,12^, ca 40 m yr^−1^ faster than the ice flow directly adjacent to NEGIS. The present-day shape and surface velocity of NEGIS are well constrained by satellite observations^11^, but much less is known about the spatial and temporal evolution of the stream, and the processes which trigger the exceptionally high flow velocities in its upstream region. Satellite and GPS measurements show that NEGIS is accelerating slightly, indicating that the ice-dynamic regime is possibly not in equilibrium^13^. Previously it was assumed that this ice stream existed in its current shape at least during most of the Holocene, and that its presence can be explained by an area of strongly enhanced geothermal heat flux at upper NEGIS^8^ causing substantial basal melting of the ice sheet. To explain the annual layer thickness along the ice column, Fahnestock and colleagues^8^ suggest a basal ice loss of ca 0.1 m per year over the last 9 kyrs. This would require an exceptionally high geothermal heat flux of 950 mWm^−2^ ^2^. However, a comparison with global geothermal heat fluxes shows that the proposed values exceed natural heat fluxes by about an order of magnitude^14^.

Here we use isochronous radar reflections as passive tracers of ice deformation to reveal the history of NEGIS over the past few thousand years, and show that the shear margins that define it in its present form in the upstream region have been active for only about the last 2000 yrs. This indicates that streaming can be triggered on short time scales, leading to abrupt ice-flow reconfigurations^15^, which is contradictory to it being triggered and sustained by a local, long-term heat flow anomaly^8,16^.

## Results and Discussion

### Radar Stratigraphy

The data presented in this study have been derived from an airborne radar survey in May 2018 using an ultra-wide-band radar system (AWI-UWB^17,18^) with an array of 8 transmitters and receivers mounted beneath the fuselage of the AWI aircraft Polar 6^19^. The layout of the survey was designed for mapping radar stratigraphy and bedrock properties in the vicinity of the EGRIP drilling camp, with an area stretching along flow from 150 km upstream of the camp to 150 km downstream. The profiles used in this study were recorded in narrow-band mode, with the frequency range set to 180-210 MHz. Due to the focus on stratigraphy, the radar lines are mostly perpendicular to ice flow in order to best reproduce the deformation pattern in the shear margins. Here we only use data from across-flow profiles (Fig. 1b). The distance between the profiles is 5 km in the central part of the survey region, in the outer area the distance is 10 km.

The ages of distinct layers in the radargrams were derived from tracing to or correlating layers at the EGRIP drill site where ages are known as a function of depth^20^. This results in a set of layers with known depositional ages. In the central profile up to 21 layers that are <8 kyrs BP old (according to EGRIP chronology) could be connected to the EGRIP site, while in the downstream profile this number was reduced to at least five. The error in dating of the reference layers is up to 200 years towards 8 kyrs BP layers for the absolute age, which is a conservative estimation^21^. For the following analysis only the relative age difference between the layers is relevant, which corresponds to the error of the ice core dating^20^. Most layers within one fold limb cannot be traced all the way to the drill site or can be recognised in the radargram at that site. Their ages are estimated by interpolation (see methods).

### Folds in ice: a record of deformation

Disturbances in radar isochrones have been conclusive to constrain temporal shifts in ice stream flow regimes in Greenland and Antarctica^15,22–24^. Modern radar systems now make it possible to investigate the processes that influence the shape of the isochrones in great detail^25–30^, and, depending on the arrangements of profiles, also in 3D^15,31^.

To analyse the overall structure of the distortion of the radar isochrones, we visualised a selected reflector as a 3D horizon^21^ (see Methods) in the abovementioned dense array of radargrams (Fig. 1b). We chose one of the deepest layers in the upper half of the ice column, deposited approximately 7150 yrs BP (EGRIP-core dating^20^) that could still be traced continuously and reliably over the entire survey area (Fig. 1d). The plot of the complete 7150 yr-layer (Fig. 1c) reveals that the ice stream has left a significant imprint on the layer shape over the entire survey area, with complexity, amplitude, and number of the folds increasing downstream.

Outside of upper NEGIS, we find upright, cylindrical folds with wavelengths up to about ten kilometres. Here we define the wavelength as the distance between two adjacent crests or troughs of folds, measured perpendicular to their hinge lines. The amplitude is then defined as the difference in depth of trough and crest. Hinge lines converge on the ice stream in a fan-like pattern (Fig. 1c), with angles relative to NEGIS increasing downstream up to ca 55° southeast of EGRIP. Here the tallest folds are found with amplitudes (*A*) that reach up to ca 500 m in the 7150 yr layer (Fig. 1d). Disturbed ice without a clear stratigraphy is brought up to over a kilometre in the cores of these folds. In the adjacent synclines, the layer of deep disturbed ice is strongly reduced in thickness.

The hinge lines of the folds can be traced from outside of the ice stream into the shear margins and, in some cases, even across the shear margin into the interior of upper NEGIS (yellow dotted line in Fig. 3). Inside the shear margin, the fold hinges rotate to almost parallel to the shear margin, their wavelengths decrease strongly to <1 km, and their amplitude is less than outside of NEGIS. It should be noticed that hinge lines are at an angle to streamlines (also called flow lines) of the current surface velocity field.

### Timescale of fold formation

Dating of the active folding process is essential to constrain fold formation but also to determine the age of the flow perturbations that lead to fold growth, in this case, the formation of upper NEGIS. Here we address this issue by presenting the results of a method, which is based on an analysis of how the fold amplitudes change with the age of the layers, and is introduced in detail in the method section. The method is so far not used in glaciology, but based on standard principles used in geology^32^.

The method to date the folding events is based on the principle that a new event leads to a steady increase in fold amplitude with depth in all existing layers. Layers deposited after the folding event are not folded. The timing of the end of the last fold event can thus be derived by determining at what age the amplitude-depth trend starts to deviate from zero. Multiple superimposed folding events result in breaks in the amplitude trend, with each break representing a folding event (Methods). Here we focus on the end of fold amplification only.

**Fig. 2:**
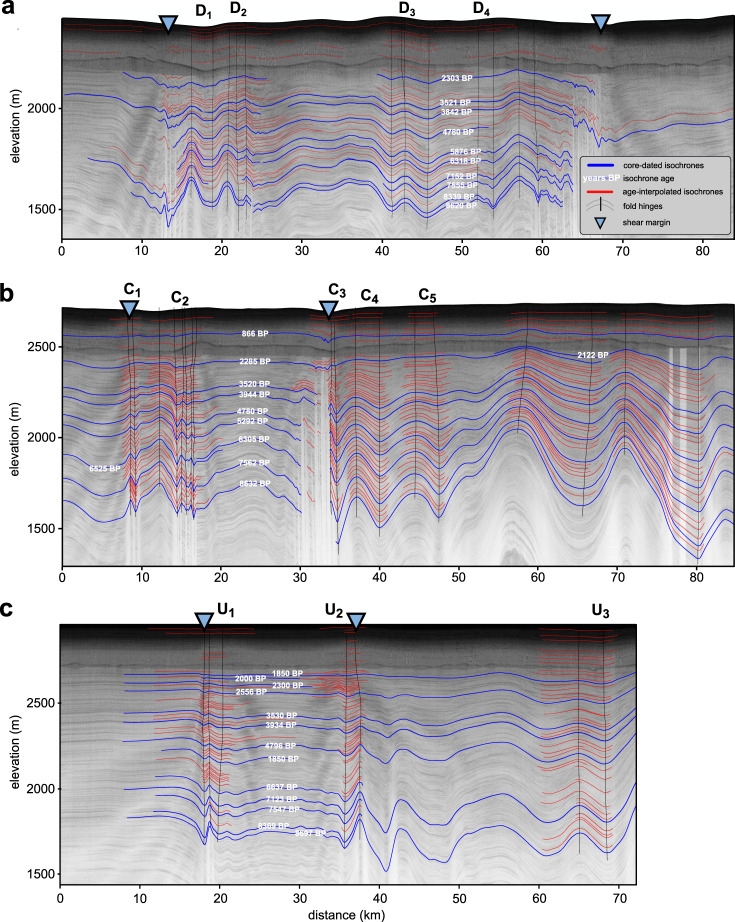
Radar sections with the picked layers for fold analysis and the reference age horizons. Black lines indicate the hinge positions or axial planes of the syn- and anticlines. Letters at the top identify folds in amplitude age plots in Fig. 3. **a** Downstream radar profile, P_D_, composed of 2 frames (20180509_01_009 and 20180509_01_010). **b** Central radar profile, P_C_, composed of 2 radar tracks (20180508_06_004 and 20180514_03_001). **c** Upstream radar profile P_U_, composed of one radar track frame (20180514_03_014). Triangles indicate the shear margins.

**Fig. 3:**
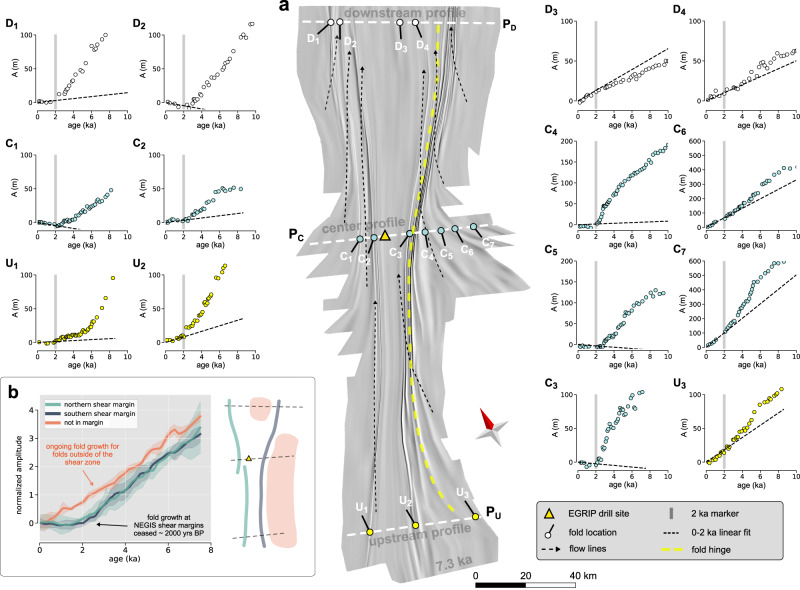
Amplitude-age graphs. Source data are provided as a Source Data file. **a** The centre of the figure shows the 3D visualisation of the 7150 years BP isochrone horizon as a hillshade to highlight the overall structure. **b** The graph on the lower left shows the mean normalised fold amplitudes vs. age for the two margins and the outside of NEGIS with shaded standard deviation. The sketch to the right shows the origin area of the mean amplitude values. Subfigures show fold amplitude vs. age at fold locations for three selected across-flow radar profiles. Black dotted lines in subfigures represent a linear fit to the data points from 2000 years BP until today and then extrapolated to older ages. Letters U, C, and D (Upstream, Central and Downstream) and numbers (increasing from left to right) indicate folds and their locations.

The relationship between depth and age is not exact, but close to linear in the Holocene ice in the study area. In Fig. 3, we therefore show amplitude-age, instead of amplitude-depth graphs, for 14 individual crest-through pairs from three radargrams perpendicular to NEGIS. One (labelled C) is at the EGRIP site in the centre of the survey area, one 130 km upstream (U), and one 90 km downstream (D) (Figs. 2 and 3 and Methods). Folds well outside of NEGIS (C_6-7_, and U_3_) and inside NEGIS (D_3-4_) show amplitudes that already start to increase from zero at the surface, which indicates currently active fold amplification. Folds inside the shear margins (D_1-2_, C_1-3_, and U_1-2_) and just adjacent to it southeast of EGRIP (C_4-5_) show very different amplitude-age trends. Here amplitudes in layers younger than 2 kyrs BP are close to zero, signifying that all these folds stopped growing by about 2 kyrs at the latest. The difference in the fold groups is clearly visible in Fig. 3b, which shows the combined-normalised amplitude-age graphs.

To compare all age-amplitude graphs in order to identify certain events in time it is necessary to normalise the amplitudes plotted on the y-axes of the inset plots of Fig. 3. For this we used the Procrustes analysis, to remove the absolute amplitude and scale. In this way common trends in the data can be made visible (details are given in the methods section). Whereas the end of folding is clearly marked, the onset of the last folding is difficult to determine. The onset of folding would be the age where the amplitude-depth trend reaches a steady slope because layers deposited towards the end of a folding event experienced less folding than those deposited at the beginning. Amplitudes in all folds start to increase steadily with depth and, hence, the age for layers older than ca 3.5-4 kyrs BP. However, some amplitude-age trends also show bends at other ages, such as ca 5-6 kyrs (U_1_, C_2_, C_3-7_, D_2_, D_4_, and U_3_) and ca 8 kyrs (D_4_ and C_4-7_). This suggests that the ice sheet here experienced multiple folding events over time, which we cannot resolve here. However, here we are concerned with the final cessation of fold amplification, which was ca 2 kyrs in and near the shear margins, while fold growth is still ongoing away from the shear margins, both outside NEGIS and in its interior.

### Folds reveal the history of NEGIS

**Fig. 4:**
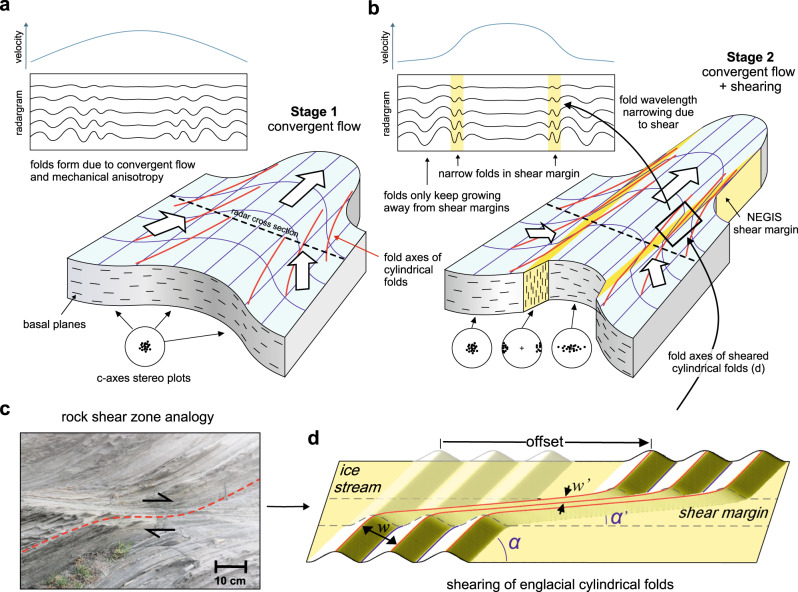
Conceptual model of fold formation. **a** Situation before localisation of strain in shear margins. Red lines indicate fold hinges, and white arrows the direction of flow. Below the block diagrams the dominating crystal fabric of the ice is illustrated as Schmidt-plots of c-axed directions. **b** After the establishment of shear zones (highlighted in yellow) and a plug-flow like regime in the central part: Inside of the shear margin the fold hinges are rotated towards parallelism with the shear margins. **c** Shear zone in schistose rock, with the foliation bending into the top-to-the-right shear zone. The picture was taken at Tudela, Cap de Creus, Spain. **d** Sketch to illustrate the three strain indicators at the shear margins: (i) reduction of wavelength, (ii) rotation and (iii) offset of fold hinges.

A conceptual model for the development of the structures in NEGIS that we see in our isochrone horizon is summarised in Fig. 4. In the upstream region of NEGIS folding was initiated before 2 kyrs. The fold hinges trend towards the exit gate of the ice stream (Fig. 4a), which is consistent with folding due to convergent flow of ice with a horizontal anisotropy, similar to the fold pattern that is observed at Petermann Glacier^31^.

The folds are sheared where they are transected by the shear margins, causing their rotation and tightening (Fig. 4d). This implies that the folds existed before the shear margins developed. Convergent flow implies the development of horizontal velocity gradients and, hence, strain-rate gradients, including zones of non-coaxial strain that are amenable to strain localisation in an anisotropic material such as ice^33,34^. Within the developing shear margins, simple shear along the vertical shear plane dominates over all other strain rate components, such as flattening due to precipitation. This leads to a rotation of the crystal basal planes to vertical and parallel to the shear margins, with concomitant geometric weakening (see the c-axes stereoplots in Fig.4b). This kind of fabric has been inferred from airborne radar measurements in the shear margins of Thwaites glacier^35^, and NEGIS^36^. Ice fabric measurements from deep ice cores at flank or dome positions with simple shear conditions towards the base of the ice sheet show that glacier ice develops a single maximum fabric with the c axis normal to the plane in which the shear is located^37^. The predominating fabric regimes are indicated in Fig. 4a,b as schematic Schmidt-plots for c-axes orientations. Numerical simulation suggests that the weakening by this change in crystallographic preferred orientation could easily be an increase in shear strain rate of one or two orders of magnitude at a given shear stress (Methods). Figure 4c shows an example of a shear zone in which folds are rotated towards parallelism with the shear zone. Here the anisotropy in metamorphic turbidites is formed by a strong alignment of the highly anisotropic mineral biotite axial planar to folds in a composite bedding and schistosity. As with the shear margins of NEGIS, the rotating anisotropy is thought to have caused localisation of deformation in shear zones^38,39^.

**Fig. 5:**
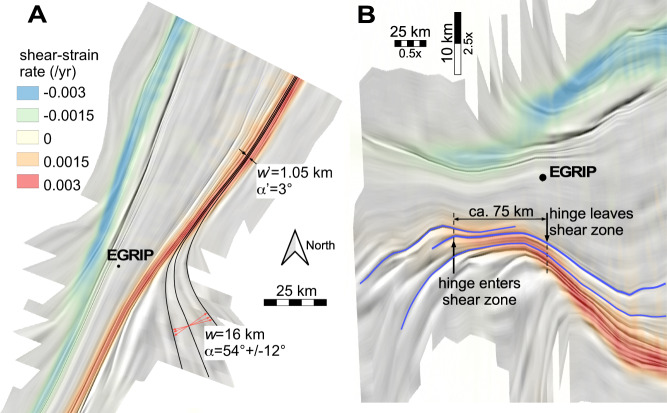
Folded isochrone horizon as a hillshade with overlay of shear strain rate. **A** Map of the folded horizon, black lines indicate traced fold hinges listed in Table 1. Location of the East Greenland Ice core Project (EGRIP) drill camp is shown as the black dot. **B** Same image, but now rotated 63° (long axis of Northeast Greenland Ice Stream, NEGIS) and then stretched 5x in the vertical direction of the rotated figure, i.e., perpendicular to the main flow direction, to highlight bends in the hinges.

Numerical simulations with anisotropic ice (see methods, Figs. 7 and 8), with the full-field ELLE+VPFFT^40–42^ code shows that folds form when basal planes are initially aligned to the shortening direction^43^. However, the shortening quickly rotates the basal planes towards parallelism with the extension direction, which causes a cessation of fold amplification. We thus explain the cessation of fold amplification in and near the shear margins by the rotation of the anisotropy that caused the shear localisation in the shear margins, as compression at a high angle to the planar anisotropy does not lead to folding or fold amplification. Where the shear margin intersects folds, the hinges of the fold trains rotate towards parallelism with the shear margins (Fig. 4d) as the ice in the ice stream is moving faster, and the fold hinges are advected with the ice flow. This is apparent in the isochrone horizon shown in Fig. 1c and the centre panel of Fig. 3, but becomes even more apparent when the image is shortened along the flow direction (Fig. 5). The observed offset of fold trains southeast of EGRIP is in the order of 75 km. The rotation of the hinges and the resulting shortening of the wavelengths of the folds (Figs. 4d and 5) provide additional indications of the amount of finite strain in the shear margins, resulting in an estimated shear strain of *γ* ≈ 18 (Methods). The total offset is the product of width of the shear margin and finite shear strain. At a width of 3-4 km, this results in a total offset of ca 55-75 km. With the current velocity difference of 40 m yr^−1^ across the shear margin, this offset would be achieved in 1375-1875 years. This is underestimated as the velocity difference across the shear margin decreases upstream. Thus, the current flow velocity is consistent with an age of about 2 kyrs BP, which is the time when fold amplification ceased in the shear margins (Fig. 3).

From the amplitude graphs and the strain measurements, we can conclude that the upstream part of NEGIS and, thus, that the present-day NEGIS as an ice stream with distinct shear margins (Fig. 4b) was fully established by about 2 kyrs BP. Geological evidence from the Northeast Greenland coast showed that the three major outlets of NEGIS (Fig.1a, the Nioghalvfjerdsfjorden Glacier (NG), the Zachariae Isstrøm (ZI), and the Storstrømmen Glacier (SG) retreated behind their current extent and advanced again at least twice during the last 45 kyrs^44^. During the Holocene Thermal Maximum (HTM) in the Early to Middle Holocene, temperatures in the Arctic were higher than today^45^. This had a large effect on Greenland ice volume and frontal positions of outlet glaciers^46^. The onset and end of this warm period were regionally different, and there is evidence from geological data that in the area of the three major outlets of the NEGIS that warming started around 8 kyrs BP and ended approximately 4 kyrs BP^45^ Accordingly, Nioghalvfjerdsfjorden Glacier was smaller than today in its extent until at least 4.6 kyrs BP^47^.

The two- with established shear margins, could be interpreted as a result of increasing discharge from the NEGIS catchment area, following the readvance of the ice front after the HTM, as ice stream activity is linked to the geometry changes of an ice sheet, with increasing intensity of streaming for higher ice volume^48^. Around 4 kyrs BP, a still distributed increase of the outflow led to a drawdown of ice from the flanks and a confluent flow regime due to the geometry of the catchment and the outlet in the northeast. Localisation due to the emerging simple shearing along the vertical plane subsequently led to localised shear and the establishment of the shear margins, which was completed by about 2 kyrs BP, with an error for the absolute age of 200 years, according to our data. Enhanced flow within the ice stream induced ice-stream normal flow in the adjacent ice sheet to compensate for the stretching inside the upstream part of the ice stream, resulting in the typical bottleneck shape of NEGIS, as the shear margins are advected towards the centre of the ice stream^49^.

Our observations and dating of folding require a paradigm change in our thinking on NEGIS and, therefore, other such ice streams. So far, NEGIS was considered a long-lived structure^4,8^ controlled by external boundary conditions, in particular high geothermal heat flux at its upstream end^8,16^. Instead, upper NEGIS is only a few thousand years old and still changing. Our results show that an ice sheet is a delicately balanced system in which the whole flow pattern can change from shallow ice-type flank flow to effective drainage systems reaching up to the divide, facilitated by shear localisation. Together with the study by Franke and colleagues^15^, we are able to draw a holistic picture of the dynamics of NEGIS-style ice streams, namely that these streams can switch between branches in their catchment area, and that such a change happened in Northeast Greenland about 2000 years ago. This time scale is another indicator that the initiation of streaming is much more likely triggered by ice sheet geometry and processes at the boundaries than local heat flow anomalies^14^. Considering that the ice sheets are now expected to experience massive changes in their boundary conditions^50^, it is imperative to include these dynamics in ice-sheet models and predictions of future sea-level rise.

## Methods

### 3D Isochrone horizon

In order to assess the distortion of the radar isochrones in terms of their deformation history to determine the dynamic setting of the ice stream, the 2-dimensional profiles have to be combined to produce a 3-dimensional model of the folded isochrone surfaces^15,31,21^. For this purpose, we picked selected internal reflections, which are detectable throughout most of the survey area. To ensure spatial continuity, we restrict our analysis to reflections from the upper half of the ice column. By manually assigning profile sections from two neighbouring lines to each other, a surface can be generated in a half-automated way. For this step of the analysis, we used the structural geology modelling software MOVE, a tool for analysis and 3-D visualisation in geosciences and previously employed to visualise folds in Northwest Greenland’s Petermann Glacier^31^ and upstream of Nioghalvfjerdsfjorden Glacier in Northeast Greenland. The isochrones are dated by tying them in the depth domain to the age dating of the EGRIP ice core^20^ and transferring age in a particular depth to the respective isochrone at that depth. The absolute age and its uncertainty of ca 90 yrs are of minor importance in our study, as we focus on the overall deformation of the initial flat shape of the considered isochrones.

### Dating of folding events

**Fig. 6:**
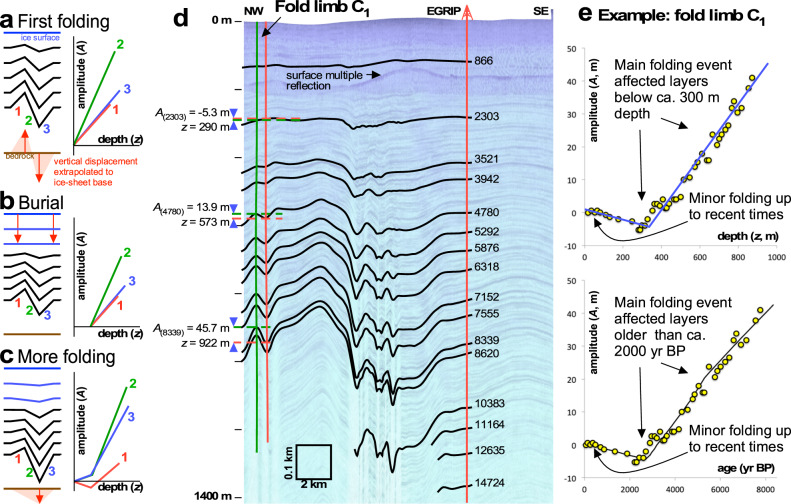
Development of fold amplitudes. **a–c** Conceptual sketches for the effect of folding on layer disturbance and development of fold amplitudes (1–3) as a function of depth. **d** Part of radargram shown in Fig. 2 with traced layers and respective folds. Shown are the uppermost 1400 m with y-axes ticks spaced 200 m. Numbers on the left show amplitudes and the corresponding reference depth for 3 examples (subscript indicates age of layer), green/red vertical lines on the left indicate the position of the fold hinges of the anti- and synclines. Red vertical line on right indicates the location of the East Greenland Ice core Project (EGRIP) core with each reference layer annotated with its age; in years BP. **e** Distribution of amplitudes of fold limb C1. The top panel shows amplitude as a function of depth and the bottom amplitude as a function of age The yellow dots indicate the data points derived from the radar layers. Red arrows indicate data points of example folds picked in **d**. The black and blue lines indicate the trend with a clear kink around 300 m depth or 2000 years of age.

The method is based on the assumption that a folding event leads to a steady increase in fold amplitude with depth in all layers older than the folding event. Thus, every change in the amplitude-age trend represents a folding event. If this event happened at time *t*_1_, layers younger than *t*_1_ are not folded, i.e., have zero amplitudes. The amplitude trend caused by a subsequent folding event at *t*_2_ will again affect all existing layers (Fig. 6). Layers between *t*_1_ and *t*_2_ in age only show the resulting amplitude-depth trend from the second folding event, while older layers show the cumulative effect of both folding events. Every change in the amplitude-age trend thus represents a folding event. It should be borne in mind that folding is not expected to occur at a distinct single point in time but over a certain period and that a change in the amplitude-age trend will be spread out over that period.

Folding of stratigraphy causes a change in vertical position of layers, either upwards (anticlines) or downwards (synclines) relative to the undisturbed layer level. This change in height increases downwards from approximately zero at the surface since the surface of ice sheets shows no or little expression of folding (less than a few tens of metres at the most in the study area). We may assume^51,52^ that the vertical flattening or thickening strain is approximately constant throughout most of the ice sheet, except in the bottom-most layers. This assumption is not dependent on the cause of the folding, as it essentially states that folding is caused by vertical movements that increase towards the base of the ice sheet. This could be because of basal melting or freezing^27,29^, variable slip rates^30^, folding due to lateral shortening^31^ or even due to flow over bedrock bumps or depressions^53^. The vertical displacement can be described with the parameter:

1 $$z=\varepsilon {z}_{0}$$

A problem is that *ε* can only be determined if the original depth *z*_0_ of a layer is known. As this is usually not the case, we can compare two adjacent vertical sections with strain *ε*_1_ and *ε*_2_. The difference *A* in depth for a layer is now given by:

2 $$A={z}_{1}-{z}_{2}$$

As long as positions 1 and 2 are close to each other (as in the hinges of a single fold), the pre-fold depths (*z*_0_) of a layer at both locations are expected to be approximately the same. This results in a linear relationship between the amplitude *A* and the mean depth of a layer <*z* > = (*z*_1_ + *z*_2_)/2:

3 $$\frac{{z}_{1}}{{\varepsilon }_{1}}=\frac{{z}_{2}}{{\varepsilon }_{2}}\iff \left\langle {z}^{{\prime} }\right\rangle=\frac{{z}_{1}+{z}_{2}}{2}=\frac{\left({\varepsilon }_{1}^{-1}+{\varepsilon }_{2}^{-1}\right)}{2\left({\varepsilon }_{2}^{-1}-{\varepsilon }_{1}^{-1}\right)}A$$

To determine the amplitude-depth curves, as many layers in a radargram as possible were manually traced for anticline-syncline pairs. Axial planes are constructed as lines that connect the fold hinges. Near the ice surface, folds may die out upwards, in which case the axial planes are extended vertically towards the surface. Depth (*z*) of a stratigraphic layer is now defined as the vertical distance between a hinge of that layer and the ice surface at the point where it is intersected by the axial plane. Comparison of independent depth determinations by two of the authors (PDB and YZ) showed differences in *z* up to 3 m, with a standard deviation of the differences of 0.9 m. For each anticline-syncline pair, referred to as a fold limb, this results in a set of *z*_*anti*_*(i)* and *z*_*syn*_*(i)* data for each layer *(i)* that was deposited at time *t(i)*. The fold-limb amplitude is now defined as *A(i)* = *z*_*syn*_*(i)*-*z*_*anti*_*(i)*, with associated mean depth <*z(i)>* = (*z*_*syn*_*(i)*+*z*_*anti*_*(i)*)/2.

Ages of layers were derived from tracing to or correlating layers at the EGRIP drill site where ages are known as a function of depth^20^. This results in a set of layers with known depositional ages. In the central profile up to 21 layers <8 kyrs BP old could be connected to the EGRIP site, while in the downstream profile this number was reduced to at least five. The error in dating of the reference layers is in the order of a few tens of years for the youngest few thousand-year-old layers, increasing to over 100 years towards 8 kyrs BP layers. Most layers within one fold limb cannot be traced all the way to the drill site or can be recognised in the radargram at that site. Their ages are estimated by interpolation, assuming that the relation between height of the layer above the bedrock (*h*) and age (*t*) is given by:

4 $$\frac{\left\langle h\right\rangle }{H}=C{e}^{-{kt}}$$

with *H* the local thickness of the ice sheet, and *C* and *k* two constants derived by fitting to the nearest dated layers above and below the layer of unknown age.

This corresponds to a Nye-type approach of age-depth relationship, where *C* and *k* are dependent on initial layer thickness and initial total ice thickness, both not well constrained in a dynamically variable setting^36^ As layer thickness are relatively constant down to layers ca 8 kyrs in age, this interpolation is close to linear. Note that mean depths for each fold were used for the interpolation. All ages are reported as before the year 2000 CE.

### Procrustes analysis

To determine common trends, we use principles of shape analysis as used in biology or anthropology. These disciplines often face the problem of comparing shapes, for example to assign or distinguish fossil remains of modern humans versus Neanderthals^54^. Here the issue is to find commonalities and difference between the shapes of the various amplitude (*A*) versus depth (*z*) or age (t) graphs (*Az or At*-graphs). We therefore employ a similar normalisation procedure, known as Procrustes analysis^55,56^, to remove scale and absolute amplitude of the *Az*-graphs. Furthermore, to be able to group folds, we need comparable data points, known as “landmarks” in geometric morphometrics. For this we first determine the amplitude *A(t,i)* of each fold limb (*i*) for a fixed series of ages (*t*), here every 100 years, by linear interpolation of the raw amplitude-age data. This was done for the period from 7500 yr BP to the present, to ensure that data for this period are available for all fold limbs. Each *Az*-graph is thus defined by 76 such landmarks.

The first step of the Procrustes normalisation is shifting all landmarks to a common reference, here the mean amplitude ( < *A(i)*>): *A’(t,i)*= *A(t,i)* - <*A(i)*>. The next step is the normalisation for scale, defined by the mean absolute shifted amplitude <|*A’(i)*|>: *A”(t,i)*= *A’(t,i)*/< | *A’(i)*|>.

Fold limbs were then divided into two groups: (i) folds in or near the shear margins, and (ii) folds inside NEGIS (only measured in the downstream section) and folds well outside of the shear margin on the southeastern side of NEGIS. Due to the very strong distortion in the southern shear margin, no folds were analysed directly inside this margin. Depths of layers are measured from the surface of the ice sheet, which means that amplitudes at the surface are zero by definition. For plotting, the normalised *A”* data are therefore shifted so that *A”*(i)=0 m. Data are plotted (Fig. 3) with 1σ error bars.

### Strain from rotation of fold hinges and the reduction of fold wavelength

Outside NEGIS fold hinges are oriented at an angle *α* relative to the shear margin (Fig. 4d). Inside the shear margin the hinges are rotated to an angle *α‘*, depending on the amount of shear strain (*γ*). The shear strain is given, assuming perfect simple shear, by:

5 $$\gamma=2{\left\{\frac{\left(1-\cos \left(2\alpha \right)\right)}{\tan \left(\alpha -{\alpha }^{{\prime} }\right)}-\sin \left(2\alpha \right)\right\}}^{-1}$$

Shear in the margins does not only rotate the fold axes, but also reduces the wavelength. Folds in the shear margins are therefore much narrower than outside the margins. We use the ratio *w*/*w’*, where *w* is the wavelength outside the shear margin and *w’* the wavelength inside the shear margin, measured in the direction perpendicular to the shear margin (Fig. 4d). Assuming simple shear we obtain:

6 $$\frac{w}{{w}^{{\prime} }}=\sqrt{1+{\gamma }^{2}}\cos \left({\tan }^{-1}\left(\gamma \right)-\alpha \right)$$

Unfortunately, there is no simple analytical solution to this equation, but the iterative solution for *γ* is trivial.

Three fold traces (shown in black in Fig. 5a) were traced. They enter the shear margin to the east of EGRIP. Their spacing *w* was determined where the fold-hinge trend *α*, relative to the shear margin, ranges from 54° to 75° and *w* from 15.9 to 16.6 km. In the marginal shear zone, the fold train narrows to approximately one km, giving *w*/*w*’ ranging from 15.9 to 16.6, depending on the value of *α* that varies within the fold train. Within the shear margin *α*‘ is about 3°. Table 1 provides the shear strain estimates, resulting in an estimated shear strain of *γ* ≈ 18.

**Table 1:** Shear strain estimates, assuming perfect simple shear and a final angle of *α*‘=3° and final wavelength of w’=1.05 km for three traced fold axes, treated as passive marker lines

fold	*α* (°)	*w*_0_ (km)	*γ*_(wavelength)_	*γ*_(rotation)_
1	54.1	16.2	19.3	18.4
2	62.1	15.9	17.5	18.6
3	74.8	16.6	16.9	18.8
mean			17.9	18.6

### Shear zone softening

The full-field ELLE + VPFFT^32,57–59^ simulation code was used to estimate the amount of weakening due to simple shearing along a vertical plane in ice with a strong crystallographic preferred orientation (CPO) in which the basal planes are initially aligned along the horizontal plane. In the semi-2D model, the material is described by 256 × 256 elements or crystallites, each with their own crystallographic orientation. C-axes were initially randomly oriented within ±5° perpendicular to the model plane. The code uses a spectral solver^38^ to determine the stress and strain-rate field compatible with the imposed boundary conditions. An average dextral simple-shear velocity field and zero strain rate in the 3rd dimension were used as boundary conditions.

**Fig. 7:**
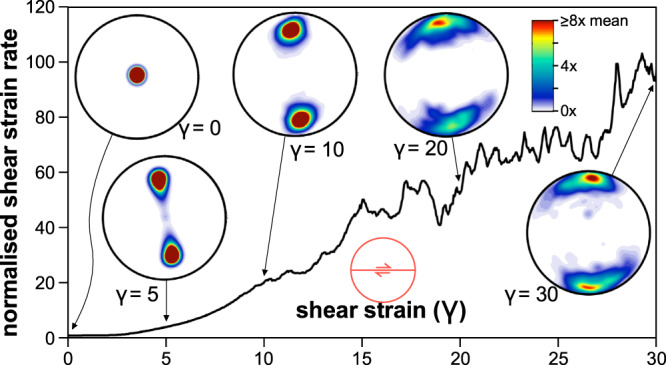
Modelling shear zone softening. ELLE + VPFFT^34,57^ results of simple shearing of ice 1 h, starting with a strong vertical point maximum of the c-axis orientations ( ±5°), a 16x weaker basal plane, and a stress exponent of 4^60^. ELLE is a numerical simulation platform for modelling microstructure in geological processes. VPFFT indicates the viscoplastic deformation code. The simulation illustrates the evolution and reorientation of the crystal-preferred orientation (CPO) from the ice sheet into the shear margin. The modelled plane is horizontal (bedrock parallel) and is then subjected to simple shear with a vertical shear plane. The graph shows the normalised shear strain rate (at constant stress) vs. shear strain as well as pole figures (lower hemisphere; classical glaciologic projection looking down a drill core into the ice) until a shear strain of γ=30. The single-point maximum orientation of c-axes first divides into two-point maxima until a shear strain of γ=10. Between γ=10 and γ=20 a transition occurs, when the two-point maxima rotate towards a broad new single maximum perpendicular to the shear plane. The total softening is about a hundred at a shear strain of γ=30, and already 20 at a shear strain of γ=10.

The code assumes that deformation is achieved by power-law slip along the basal, prismatic and pyramidal crystallographic planes of ice 1 h, using a stress exponent^60^ of 4. The strong anisotropy of ice 1 h is incorporated by setting the friction parameter or critical resolved shear stress 16x lower for the basal plane than for any other slip planes. Ice deforming by basal slip only is thus 16x weaker than ice deforming by slip along the other planes at a given strain rate. Von Mises stress and strain-rates are used to describe the bulk strength of the material as a function of strain (Fig. 7). Each step, the velocity field is used to calculate and update the lattice rotation in each element. The CPO is visualised (Fig. 7) by plotting the c-axes’ ODF (orientation density function) which represents the volume fraction of crystallites with a certain orientation in a lower hemisphere stereographic projection (using the texture analysis software MTEX^61^).

### Folding of anisotropic ice

**Fig. 8:**
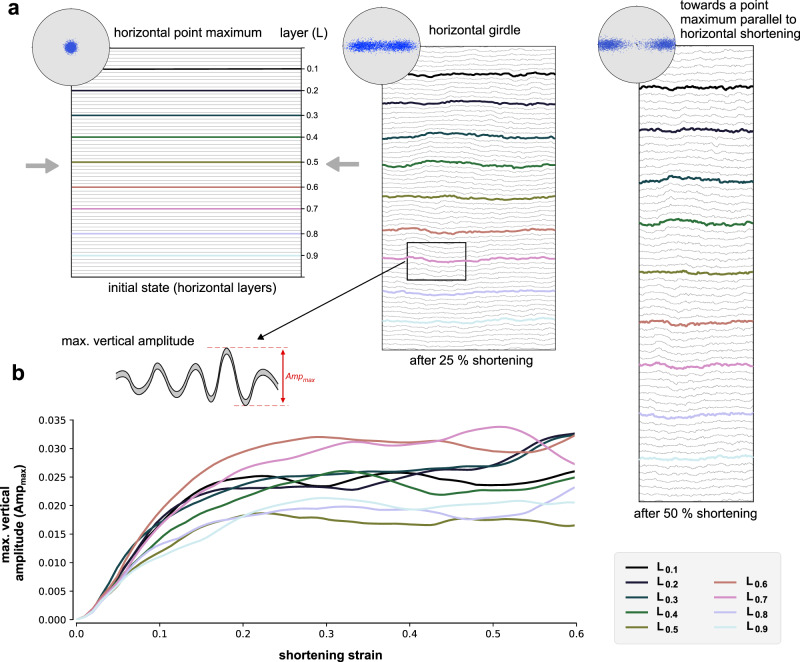
Result of numerical modelling of folding in anisotropic ice (ELLE + VPFFT). ELLE is a numerical simulation platform for modelling microstructure in geological processes. VPFFT indicates the viscoplastic deformation code. **a** Whole model at three stages of plane-strain horizontal shortening (0, 25 and 50%) with passive marker lines that illustrate the folding of originally horizontal, mechanically passive layers (L). Insets show the distribution of c-axes that are perpendicular to the easy glide basal planes of ice. Projection is looking down from the top of the model, parallel the vertical extension direction. C-axes are thus initially aligned parallel to the extension direction with a standard deviation of ±5°. Folding of the aligned basal planes leads to the formation of a girdle distribution with two point maxima that move to the shortening direction with increasing strain. **b** Graph of the maximum vertical amplitude of nine equally spaced, initially horizontal passive marker lines, highlighted in colour in (**a**). The maximum vertical amplitude is the difference between the highest and lowest point along a marker line (inset). Fold amplification ceases at about 25% shortening when the initial strong alignment of c-axes is converted to a partial girdle.

In another ELLE + VPFFT simulation, similar to that to model the shear softening, we investigated the folding of passive lines parallel to the shortening direction in pure-shear shortening. Modelling code and all settings were identical to those described for modelling shear softening (see above), except for the boundary conditions. The initially square model was deformed under plane-strain, pure shear velocity boundary conditions with horizontal shortening and vertical extension in steps of 2% shortening. C-axes (normal to the easy-glide basal plane) were initially aligned parallel ( ± 5° standard deviation) to the vertical extension direction. The deformation of initially horizontal passive marker lines was traced, using the calculated velocity field for each step, to reveal folding induced by the deformation of the anisotropic material. Figure 8a shows that folds form and that the CPO evolves from an initial point maximum to a girdle with two maxima, and finally towards a point maximum parallel to the shortening direction. Fold amplitudes were determined by taking the distance between the highest and lowest point along one folded marker line. Figure 8b shows that active fold amplification ceases from about 25% shortening.

### Supplementary information

Description of Additional Supplementary Information

Supplementary Dataset 1

## Data Availability

The ice thickness data of the EGRIP-NOR-2018 survey and refined bed topography^9^ is available at: https://doi.pangaea.de/10.1594/PANGAEA.907918 The complete radar data set will be additionally made available as part of a data collection for Northern Greenland. The GICC05-EGRIP-1 timescale for the EGRIP ice core^20^ can be obtained here: 10.1594/PANGAEA.922139. Amplitudes, depths and age of the picked isochrones for the dating of the folds are available as Extended Data Material. The EGRIP-NOR-2018 airborne radar profiles are available from PANGAEA: 10.1594/PANGAEA.928569^62^. The three-dimensional 7.3 ka stratigraphic horizon of NEGIS is available from PANGAEA: https://doi.pangaea.de/10.1594/PANGAEA.954991^63^. The data points for the age-depth graphs in Fig. 3 are available in the source data file in Supplementary Data Set 1
