# Supplementary material for: Shear margins in upper half of Northeast Greenland Ice Stream were established two millennia ago
Source: Nat Commun. 2024 Feb 8;15:1193. doi: 10.1038/s41467-024-45021-8 (PMC10853536; doi:10.1038/s41467-024-45021-8)
Supplement: Supplementary file 1 — Description of Additional Supplementary Information [file 41467_2024_45021_MOESM1_ESM.pdf]

Inventory of Supporting Information

File Name: Supplementary Data 1

Description:
This excel spread sheet contains the data points shown in the amplitude-age graphs shown in figure 3. There is one data sheet for each of the analyzed features:

- U1, U2, and U3 for the upstream radar profile P_U
- C1, C2, C3, C4, C5, C6, and C7 for the central radar profile P_C
- D1, D2, D3, and D4 for the downstream radar profile P_D
